# TBC of the Thoracic Wall with Fistulisation through the Breast

**DOI:** 10.5334/jbr-btr.924

**Published:** 2015-12-30

**Authors:** S. Van Peer, I. Wittevronghel, K. A. Vanwambeke, F. M. Vanhoenacker, N. Willers

**Affiliations:** 1 Department of Gynaecology, AZ Sint-Maarten Duffel-Mechelen, BE; 2 Department of Radiology, AZ Sint-Maarten Duffel-Mechelen, BE; 3 Department of Radiology, Antwerp University Hospital, Wilrijkstraat 10, B-2650 Edegem, BE; University of Ghent, Faculty of Medicine and Health sciences, De Pintelaan 185, B-9000 Ghent, BE

**Keywords:** breast, tuberculosis, fistulisation

A 53-year-old North African woman presented with a longstanding history of ulcerations of the right breast. Physical examination showed (Fig. 1 subfigure) an ulcer of 1.5 cm in the outer inferior quadrant, another smaller areolar ulcer and a discharging sinus tract in the inframammary sulcus. Apart from female genital mutilation, her past medical history was negative. Laboratory work up was essentially normal, culture of the ulcers were taken. Mammography showed infra-areolar skin retraction, associated with irregular, high density distortion of the breast tissue. Ultrasound (Fig. 1) revealed communicating sinus tracts coming from an intercostal mass with central necrosis. Mobile internal echoes were suggestive for abscess formation and a truecut biopsy was taken. An important granulomatous inflammatory pattern and fibrosis were found. Axillary lymphadenopathy was present.

**Figure 1:**
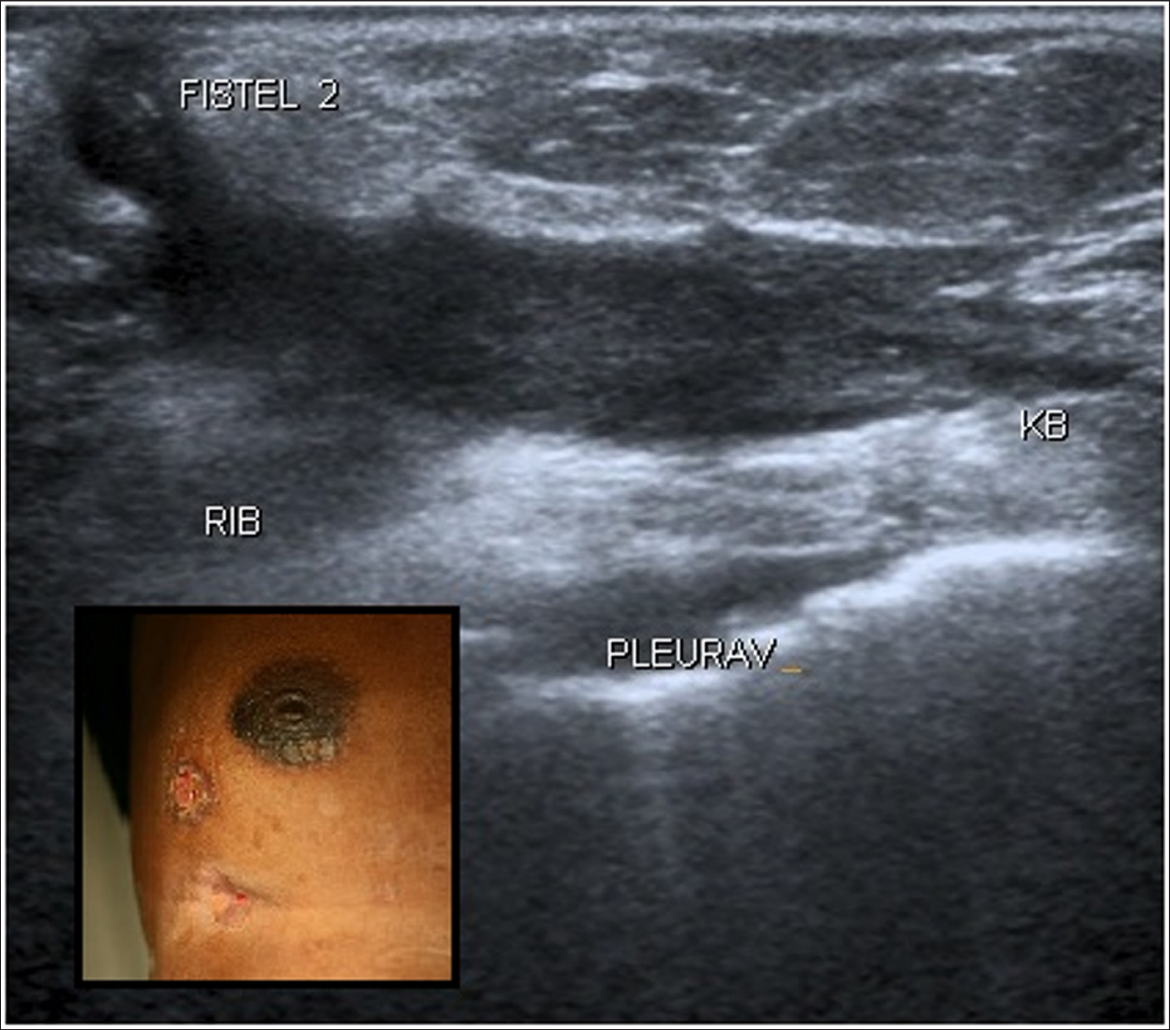
Ultrasound of the breast. Subfigure: Clinical presentation.

Subsequent CT-scan showed the destruction and scalloping of the fourth rib and local pleuritis. T1 weighted MRI with IV-injection of gadolinium (Fig. 2) nicely delineated the contours of the abscess located at the thoracic wall beneath the pectoralis major muscle with intercostal extension. Fig. 2 subfigure nicely demonstrated a long sinus tract to the skin.

**Figure 2:**
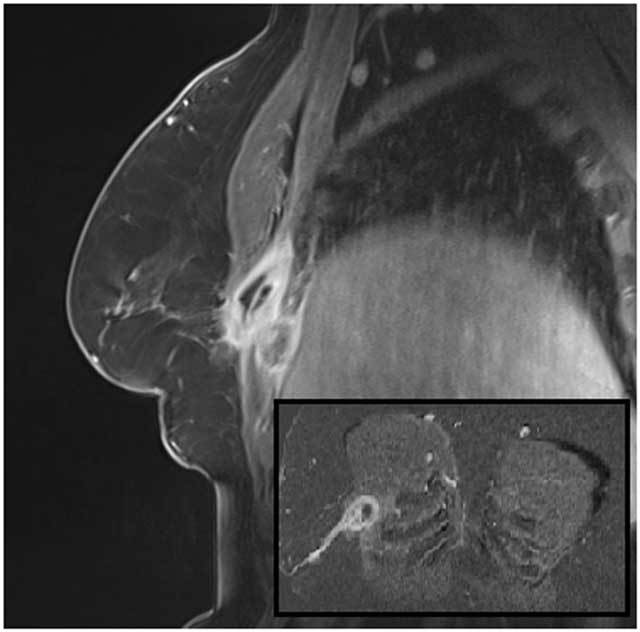
MRI sagittal reconstruction with IV gadolinium contrast. Subfigure: coronal MIP reconstruction.

A Mantoux tuberculin skin test and polymerase chain reaction (PCR) were positive for Mycobacterium tuberculosis. The diagnosis of tuberculosis (TB) was confirmed and the patient started with anti-tuberculous therapy, consisting of a three-month regimen of quadruple therapy (rifampicin, isoniazid, ethambutol and pyrazinamide), followed by a nine-month double therapy.

## Comment [1]

Despite the fact that tuberculosis (TB) remains one of the world’s deadliest communicable diseases, TB of the breast is a rare disease. TB accounts for approximately 4% of all breast lesions in TB endemic countries and less than 0.1% of all breast lesions in Western countries, nevertheless re-emerging and HIV is a major risk factor [1]. TB should always be considered if there is chronic ulceration of the breast, and the origin can be as well the breast parenchyma or the thoracic wall.

Primary tuberculous mastitis (TBM) is confined solely to the breast, most often affecting the upper outer quadrant. Secondary TBM is seen with coexisting TB and can be caused by spread from contiguous structures, as in our case. An extramammary source is identified in less than 15% of cases [1]. TBM is classified into three main types: nodular, disseminated and sclerosing [1].

1. *Nodular*: formation of a non-caseating granuloma producing a well-defined, smoothly marginated nodule due to low grade infection. Ultrasound features can mimic a fibroadenoma. If there is abscess formation, perilesional oedema or scarring, the nodule is irregular in outline and may mimic cancer.
2. *Disseminated*: multiple foci of TB intercommunicate and develop into abscesses in case of more virulent infection. On ultrasound, the abscess is characterized by a heterogeneous, hypoechoic mass with irregular margins and posterior acoustic enhancement. Mobile internal echoes are suggestive for abscess formation. On mammography, the centre of the lesion is less dense than the periphery due to fluid breakdown centrally, which may mimic an inflammatory cancer with skin thickening.
3. *Sclerosing*: the dominant feature is fibrosis. On mammography the breast becomes denser. When the Cooper’s ligaments become involved by the fibrotic process, there is retraction of the skin, distortion and atrophy of the breast similar to malignancy.

Lymph node enlargement is frequently present. The mammogram is often nonspecific. Ultrasound can differentiate solid from cystic lesions, identifying nodules masked by the coarse stroma and assessing the lymph node status [1]. MRI has not been extensively used in TBM. A breast abscess will demonstrate a ring like bright signal intensity on T2-weighted images [1]. In post-gadolinium sequences, there is a non-specific enhancement [1]. MRI is the preferred technique to document extramammary intrathoracal extent of the disease.

In the differential diagnosis other mycotic or pyogenic abcesses and inflammatory breast cancer should be considered.

## Competing Interests

The authors declare that they have no competing interests.
